# Opioid Use After First Opioid Prescription in Children With Sickle Cell Disease

**DOI:** 10.1001/jamapediatrics.2023.6500

**Published:** 2024-02-19

**Authors:** Angela B. Snyder, Mei Zhou, Brandon K. Attell, Lindsey L. Cohen, Sierra Carter, Fiona Bock, Carlton Dampier

**Affiliations:** 1 Georgia Health Policy Center, Georgia State University, Atlanta; 2 Psychology Department, Georgia State University, Atlanta; 3 Department of Pediatrics, Emory University School of Medicine and Aflac Cancer and Blood Disorders Center, Children’s Healthcare of Atlanta, Atlanta, Georgia

## Abstract

This cohort study assesses the level of opioid use, number of vaso-occlusive crises, and days’ supply of opioids among opioid-naive pediatric patients.

Vaso-occlusive crises (VOCs) are recurrent, unpredictable bouts of acute pain starting in childhood in those with sickle cell disease (SCD).^1^ These VOCs are often treated at home with analgesics, including opioids, prescribed in acute hospital settings or by pediatric hematologists. The opioid crisis has raised concerns about opioid prescribing’s role in misuse and overdose in pediatric patients.^2^ Some studies suggest that opioid-naive patients in emergency departments (EDs) being prescribed opioids for acute pain are at increased risk for additional opioid use^3^; however, there are sparse data regarding actual rates of opioid prescribing in SCD.^4^ We examined patterns of opioid use emerging within 3 years after the first filled prescription in opioid-naive children with SCD and described demographic factors of opioid use.

## Methods

This retrospective cohort study used Medicaid enrollment and claims data from the 2011-2019 Georgia Sickle Cell Data Collection (SCDC) program. The SCDC included 2565 patients with confirmed or probable SCD diagnosis^5^ aged 1 to 15 years by 2016, who had Medicaid coverage. Eligible children filled at least 1 opioid prescription between ages 0 to 9 years after 1 year without opioid prescriptions. Children were followed up for 3 years from 2012 to 2019; those missing more than 6 months of Medicaid coverage during follow-up were excluded. The Georgia State University Institutional Review Board approved the study and waived informed consent under secondary research exemption. We followed the STROBE reporting guideline.

Primary outcome for the initial group-based trajectory modeling was days’ supply of opioids per quarter over 3 years. Outcome was reported as mean (SD) or median (IQR) and categorized by first (25%) and third (75%) quantiles of days’ supply over 3 years. Descriptive variables included age, sex, SCD genotype, opioid type, and number of VOCs. All variables were defined using Medicaid data, except laboratory-confirmed SCD genotype; only VOCs treated in medical settings (eg, EDs, hospitals) were counted. Pearson correlation coefficient assessed correlation between VOCs and days’ supply of opioids. Total prescriptions, days’ supply per prescription, and whether prescription was filled within 5 days of the VOC were also ascertained.

Two-sided *P* < .05 indicated statistical significance. Data analysis was performed between January and November 2023 using SAS 9.4 (SAS Institute).

## Results

Among 725 children (mean [SD] age, 4.6 [2.5] years; 344 female [47.4%], 381 male [52.6%]) who received an initial opioid prescription, only 1 pattern of low opioid use emerged. Four hundred twenty-one patients (58.1%) had confirmed hemoglobin SS or hemoglobin S β^0^-thalassemia. Descriptive statistics are reported in the Table. Mean (SD) days’ supply of opioids over 3 years was 30.0 (41.3), with a median (IQR) of 17.0 (9.0-37.0). During follow-up, 171 patients (23.6%) had 0 VOCs, 330 (45.5%) had 1 to 3, and 224 (30.9%) had over 3. Correlation between number of VOCs and days’ supply was *r* = 0.58 (*P* < .001) (Figure). Of 3215 prescriptions, 818 (25.4%) were filled within 5 days of a hospitalized VOC. Regardless of when filled, median (IQR) days’ supply per prescription was 5.0 (4.0-8.0).

**Table.** Demographic and Clinical Characteristics by Days’ Supply of Opioid From 2012 to 2019

Characteristics	Total No. (%) (n = 725)	Days’ supply of opioid, No. (%)		
		1-9 (n = 193)	10-36 (n = 344)	>36 (n = 188)^a^
Age at first opioid prescription, y				
1	81 (11.2)	14 (7.3)	38 (11.0)	29 (15.4)
2	104 (14.3)	31 (16.1)	43 (12.5)	30 (16.0)
3	101 (13.9)	31 (16.1)	44 (12.8)	26 (13.8)
4	86 (11.9)	18 (9.3)	48 (14.0)	20 (10.6)
5	88 (12.1)	20 (10.4)	48 (14.0)	20 (10.6)
6	66 (9.1)	18 (9.3)	34 (9.9)	14 (7.4)
7	70 (9.7)	24 (12.4)	31 (9.0)	15 (8.0)
8	65 (9.0)	21 (10.9)	25 (7.3)	19 (10.1)
9	64 (8.8)	16 (8.3)	33 (9.6)	15 (8.0)
Race and ethnicity^b^				
Black	684 (94.3)	180 (93.3)	322 (93.6)	>177 (>94.1)
Other or unknown^c^	41 (5.7)	13 (6.7)	22 (6.4)	<11 (<5.9)
Sex				
Female	344 (47.4)	86 (44.6)	165 (48.0)	93 (49.5)
Male	381 (52.6)	107 (55.4)	179 (52.0)	95 (50.5)
SCD genotype				
Hb SS or Hb S β^0^-thalassemia^d^	421 (58.1)	91 (47.2)	196 (57.0)	>127 (>67.5)
Hb S β^+^-thalassemia	49 (6.8)	14 (7.3)	24 (7.0)	11 (5.9)
Hb SC	171 (23.6)	49 (25.4)	83 (24.1)	39 (20.7)
Other or unknown^e^	84 (11.6)	39 (20.2)	41 (11.9)	<11 (<5.9)
Opioid type at first prescription				
Codeine	192 (26.5)	62 (32.1)	86 (25.0)	44 (23.4)
Hydrocodone or other	533 (73.5)	131 (67.9)	258 (75.0)	144 (76.6)
No. of VOC events/3 y				
0	171 (23.6)	87 (45.1)	70 (20.3)	14 (7.4)
1-3	330 (45.5)	88 (45.6)	186 (54.1)	56 (29.8)
>3	224 (30.9)	18 (9.3)	88 (25.6)	118 (62.8)
VOC events/3 y, median (IQR), d	2 (1.0-4.0)	NA	NA	NA
VOC events/3 y, mean (SD), d	3 (3.5)	NA	NA	NA
Total supply/3 y, median (IQR), d	17.0 (9.0-37.0)	NA	NA	NA
Total supply/3 y, mean (SD), d	30.0 (41.3)	NA	NA	NA

Abbreviations: Hb, hemoglobin; NA, not applicable; SCD, sickle cell disease; VOC, vaso-occlusive crisis.

^a^ Cell sizes smaller than 11 were suppressed according to Centers for Medicare and Medicaid Services policy.

^b^ Race and ethnicity were self-reported and obtained from Medicaid claims and enrollment data. Only Black patients are included in the table due to small sizes for other racial and ethnic categories. Ethnicity is a separate variable in Medicaid claims and enrollment data but is missing from too many records to report accurately.

^c^ Other race and ethnicity included Asian and White.

^d^ Sickle cell anemia: Hb SS or Hb S β^0^-thalassemia.

^e^ Other SCD genotype included compound heterozygous forms of SCD.

**Figure.**
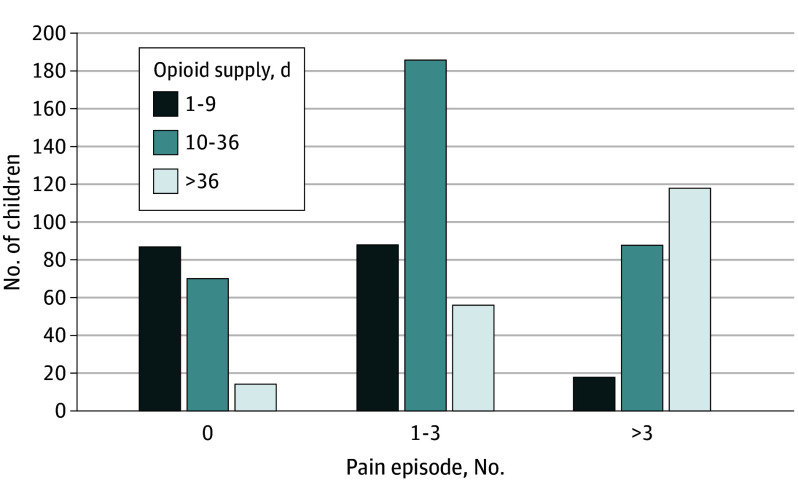
Children With Sickle Cell Disease Categorized by Pain Episodes and Days’ Supply of Opioids Over 3 Years

## Discussion

In opioid-naive children with SCD, no concerning patterns of long-term or increasing use of opioids were identified within 3 years after their first opioid prescription. Generally, patients filled less than a 30-day supply despite most having at least 1 VOC needing medical attention during follow-up. The SCD guidelines recommend rapid opioid treatment for VOC-related pain.^6^

While the number of VOCs was associated with days’ supply, only 25.4% of opioid prescriptions were filled within 5 days of VOC hospitalization to address a need for continued analgesics after discharge. This finding suggests that most prescriptions are written in outpatient settings by SCD specialists to help manage severe pain at home. Future research should examine whether low opioid use reflects effective nonopioid pain management strategies or highlights an unintended and potentially harmful treatment access problem secondary to the opioid epidemic. A study limitation is the focus on patients with Medicaid, potentially restricting generalizability to patients with commercial insurance.

## Notes

Supplement.
Data Sharing Statement
