# Supplementary material for: Opioid Use After First Opioid Prescription in Children With Sickle Cell Disease
Source: JAMA Pediatr. 2024 Feb 19;178(4):408–10. doi: 10.1001/jamapediatrics.2023.6500 (PMC10877502; doi:10.1001/jamapediatrics.2023.6500)
Supplement: Supplement. — Data Sharing Statement [file jamapediatr-e236500-s001.pdf]

Data Sharing Statement

Snyder. Opioid Use After First Opioid Prescription in Children With Sickle Cell Disease. *JAMA Pediatr*. Published February 19, 2024. doi:10.1001/jamapediatrics.2023.6500

Data
Data available: No

Additional Information
Explanation for why data not available: Aggregate level data is available, but individual-level data can not be made available due to data sharing restrictions with data stewards.
